# Multielement Ring Array Based on Minute Size PMUTs for High Acoustic Pressure and Tunable Focus Depth

**DOI:** 10.3390/s21144786

**Published:** 2021-07-13

**Authors:** Eyglis Ledesma, Iván Zamora, Arantxa Uranga, Núria Barniol

**Affiliations:** Departament d’Enginyeria Electrònica, Universitat Autónoma de Barcelona, 08193 Bellaterra, Spain; eyglis.ledesma@uab.es (E.L.); ivan.zamora@uab.es (I.Z.); arantxa.uranga@uab.es (A.U.)

**Keywords:** PMUTs, annular array, ring array, ultrasound, AlN, ultrasound imaging, pulse-echo, PMUT-on-CMOS

## Abstract

This paper presents a multielement annular ring ultrasound transducer formed by individual high-frequency PMUTs (17.5 MHz in air and 8.7 MHz in liquid) intended for high-precision axial focalization and high-performance ultrasound imaging. The prototype has five independent multielement rings fabricated by a monolithic process over CMOS, allowing for a very compact and robust design. Crosstalk between rings is under 56 dB, which guarantees an efficient beam focusing on a range between 1.4 mm and 67 µm. The presented PMUT-on-CMOS annular array with an overall diameter down to 669 µm achieves an output pressure in liquid of 4.84 kPa/V/mm^2^ at 1.5 mm away from the array when the five channels are excited together, which is the largest reported for PMUTs. Pulse-echo experiments towards high-resolution imaging are demonstrated using the central ring as a receiver. With an equivalent diameter of 149 µm, this central ring provides high receiving sensitivity, 441.6 nV/Pa, higher than that of commercial hydrophones with equivalent size. A 1D ultrasound image using two channels is demonstrated, with maximum received signals of 7 mVpp when a nonintegrated amplifier is used, demonstrating the ultrasound imaging capabilities.

## 1. Introduction

Ultrasound is widely used as an excellent noninvasive diagnostic tool for nondestructive testing and medical imaging. Nowadays, small ultrasound probes are being extensively pursued in areas such as in-body controllable catheter-based imaging for intravascular imaging [1,2,3,4], specific heat treatments based on high-intensity focused ultrasound (HIFU systems) [5,6], or brain stimulation for in vivo experiments [7,8]. In addition to these applications, power-free implantable prostheses for sensing biological parameters in animals or humans are also a rapidly evolving field of research towards digital medicine and in view of elderly population growth. Recently, powering and data transfer for these devices by ultrasound is being studied and presented as one of the alternatives in comparison with radiofrequency and inductive links [9,10,11].

For all these cases, tiny ultrasound transducers with very controllable and tunable focus depth are required. Among the different ultrasound probes, annular rings provide by layout this capability. Their dynamic focusing along the axial direction and their symmetry produce an acoustic pattern with a high spatial resolution (lateral and axial) and high levels of output pressure which have been efficiently used in most of the above-mentioned applications [1,3,4,5,6,8,9].

Annular rings for ultrasound systems have been fabricated with bulk piezoelectric materials in their thickness vibration mode. However, their complex fabrication process would limit their use in advanced ultrasound systems which require minute sizes, high reproducibility, cost-efficiency, and low power consumption [12,13]. Advances in the fabrication of micromachined ultrasonic transducers using MEMS-based technologies, either capacitive (CMUTs) or piezoelectrical (PMUTs) with the capability of direct integration with CMOS, in a batch processing approach, decrease manufacturing cost and allow a reduction in size and increase in compactness of the overall ultrasound system [12,14,15]. Ring-shaped CMUT arrays have been widely described in the literature [3,4,5,12,14]. However, their eligibility can be affected by the high power consumption needed in the CMUT transducers. On the contrary, monolithic ultrasound systems based on PMUTs open the way to power-efficient single-chip ultrasound systems [15,16].

In PMUTs, the ultrasound wave is produced due to the flexural movement of the membrane, and its operational frequency will depend on both the different thicknesses of the material and the size of the transducer, allowing in this way different operation frequencies using different device layouts but with the same technological approach. Few examples of continuous annular rings under this flexural approach have been reported [6]. However, continuous annular array fabrication could be a challenge due to its dimensional constraints. In order to avoid this and increase the achievable output pressure at the acoustic axis, a finite number of small ultrasound transducers configured in an annular ring can be used (see for example [1,17]). The main advantage in this multielement ring configuration is given by the capability to decouple the acoustic wave frequency, due exclusively to the single element, from the overall ring diameter, keeping a longer-range tunable focus depth, as is shown in Section 2. Taking these demands into consideration, we have designed a multiple concentric annular ring ultrasonic transducer composed of multiple high-frequency piezoelectrical micromachined ultrasound transducers (PMUTs) capable of being monolithically integrated with CMOS technology [16]. The fabricated multielement ring ultrasound array provides high accurate tunable focus depth, high acoustic output pressure, and minute size which is operable in the 10 MHz frequency range in a liquid environment and can be a candidate for the above-mentioned applications. In addition, we have characterized the central ring of the array as an ultrasonic receiver, showing higher receiver sensitivity with smaller spatial averaging effects than commercial hydrophones with similar size.

This paper is organized into four sections: Section 2 explains the multielement ring array design and its benefit over the continuous ring array using analytical equations and FEM simulations (COMSOL Multiphysics); predictions of the acoustic performance with Field II software are also provided. Section 3 shows the experimental results, including the electrical and acoustic characterization and line-scan ultrasound imaging as a demonstration of the full transceiver ultrasound system. Finally, Section 4 concludes the paper.

## 2. Materials and Methods

### 2.1. Multielement Ring Array Design

As already explained, the main advantage of an annular ring is its capability to be axially focused; focus depth tunability, beam diameter at the focus point, and range of achievable pressure are its main parameters. The focus depth, *F_d_*, is defined by Equation (1), where *S* is the transducer area (whatever its shape), *λ* is the wavelength, *f* is the frequency, and *c* is the sound velocity in the propagation medium [18]. It is evident that transducers with the same area will produce a greater focus depth with higher frequency.

(1) $$F_{d}=\frac{S}{4\lambda }=\frac{S\times f}{4\times c}$$

**Figure 1:**
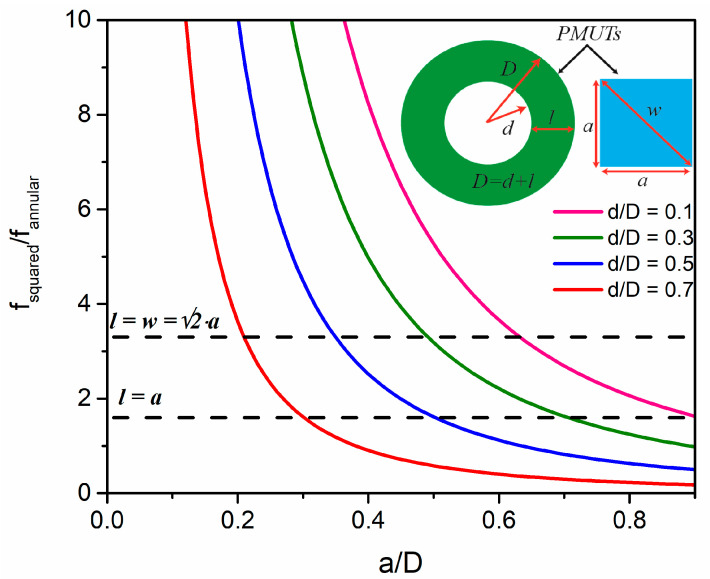
Theoretical frequency ratio, *f_s_/f_a_*, from two single elements of an annular array and a multielement multiring array with the same area according to Equation (4). Inset: Only one annular ring and one single square PMUT from the annular and multielement ring arrays are shown.

For the design of an efficient annular ring, the first question is how we can achieve the highest frequency using the same transducer area, which will give us the greatest focus depth. In our approach, we must consider flexural resonators as we are using a MEMS-based approach with AlN as the piezoelectrical material. We will analyze this focus depth capability considering a continuous annular ring array in comparison with a multielement annular ring array. Figure 1 top inset shows a conceptual schematic of a single element from a continuous annular ring array (green) and a single element (squared PMUT) that will form a multielement ring array, (blue). Here *2D*, *2d*, and *l* are the outer diameter, inner diameter, and width of each ring in the annular array; *a* and *w* are the side and diagonal of each square PMUT in the multielement array.

First, the resonance frequency for the first flexural mode for the annular ring array is giving by Equation (2), where *λ_ij_annular_* is a dimensionless parameter associated with the vibration mode (*i*, *j*), *D_r_* is the flexural rigidity, and *µ* is the mass per unit area [19]. Intuitively, higher resonance frequencies could be obtained with smaller devices, but the parameter *λ_ij_annular_* is dependent on the ratio of *d/D* and boundary conditions, so in consequence, the same frequency can be obtained for rings with the same width (no matter how big the outer diameter is), even in the case of flexural resonant rings.

(2) $$f_{a}=\frac{\lambda_{ij\_annular}{}^{2}}{2\pi D^{2}}\sqrt{\frac{D_{r}}{\mu }}\;i=1,2,\ldots j=1,2,\ldots$$

Second, for a multielement ring array, the resonance frequency is fixed by the individual PMUTs, defined by Equation (3) for the first flexural mode considering square PMUTs [20].

(3) $$f_{s}=\frac{35.99}{2\pi a^{2}}\sqrt{\frac{D_{r}}{\mu }}$$

Using Equations (2) and (3), the ratio *f_s_/f_a_* for the same layer stack is given by Equation (4) and computed in Figure 1 for different *d/D* ratios considering clamped boundaries where the parameter *λ_ij_annular_*^2^ has been extracted from Table 11-2 in [19]. The multielement ring array achieves frequencies higher than the annular array when the PMUT side is much smaller than *D*. Dotted lines show the case when the ring width is equal to *a* (minimum side of the squared PMUT) or *w*, giving for all *d/D* ratios an improvement in multielement multiring frequency of 1.6× or 3.3×, respectively. This increase in the resonance frequency is translated into a higher focal length.

(4) fsfa=35.99(aD)2×λij_annular2 i=1,2,…j=1,2,…

**Figure 2:**
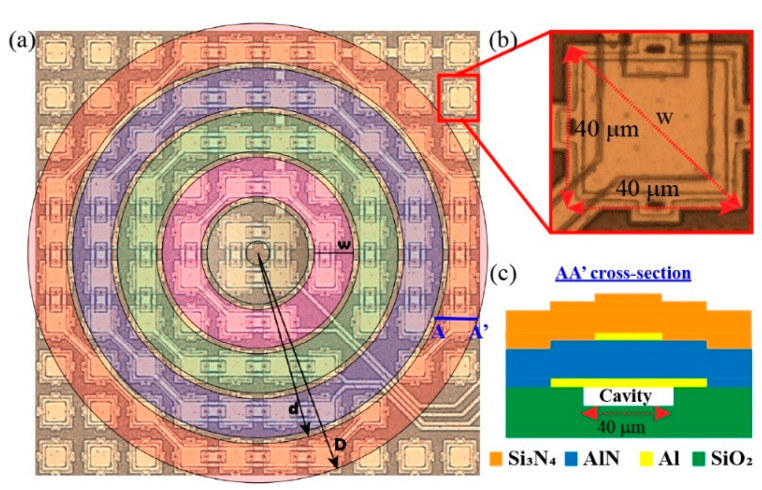
(**a**) Optical image of the multielement ring array transducer and schematic representation of the continuous rings over it; (**b**) zoom of the individual 40 µm AlN PMUT; (**c**) AA’ cross-section of AlN-PMUT.

Figure 2a shows an optical image of the proposed multielement ring array ultrasound transducer. The PMUTs are arranged in irregular polygons that are connected through the top electrode forming five concentric channels. The bottom electrode is common for all PMUT devices, and the gap between consecutive elements is 25 µm.

Every single element consists of a squared AlN-PMUT with a 40 µm side, fabricated using the MEMS-on-CMOS SilTerra technology [16,21]. The top electrode size was optimized to maximize the membrane velocity and consequently the output pressure. A 1.3 μm AlN piezoelectric material was deposited by physical vapor deposition (PVD) and sandwiched between two Al electrodes (0.35 μm thickness top electrode and 0.4 μm thickness bottom electrode). A 1.5 μm Si_3_N_4_ layer was deposited with a low-temperature plasma-enhanced chemical vapor deposition (PECVD) process; it acts as the elastic layer and seals the cavity. Figure 2c shows a cross-section profile (AA’) of the square PMUT device, and Table 1 summarizes the material properties used in FEM COMSOL simulations and the principal geometric dimensions.

The first mode shape and its resonance frequency for a square 40 µm AlN PMUT were obtained in COMSOL Multiphysics; the value of 27.7 MHz was given, which is close to the value computed using Equation (3), 27.8 MHz. Dynamic simulations in a liquid environment (Fluorinert, FC-70, with a density *ρ* = 1940 kg/m^3^ and the sound velocity c = 685 m/s) give a maximum displacement at 13 MHz.

In order to compare the performance of this multielement ring array transducer with an equivalent continuous concentric ring, we considered the circles that surround the single-PMUT elements. The shape of each ring is close to an irregular polygon, and it is not possible to obtain rings that include all PMUTs of the polygon exactly. Figure 2a shows in different colors the geometric representation corresponding to each continuous ring, where *D* and *d* are the outer and the inner radii, respectively, and *w* is the width. The values of *D* and *d* were computed trying to include the highest number of PMUTs of each irregular polygon into the continuous ring. The width, *w*, corresponds to the PMUT diagonal (the furthest PMUT point inscribed in the continuous ring) and is 56.6 µm (√ (40 µm)^2^ + (40 µm)^2^) (see Figure 2b). A gap of 8.4 µm between rings is obtained. Decreasing the ring width to *w = a*, higher resonance frequency for the continuous ring can be achieved at the expense of not including the largest number of PMUTs within it. Table 2 summarizes the computed dimensions taking into account all these considerations.

**Figure 3:**
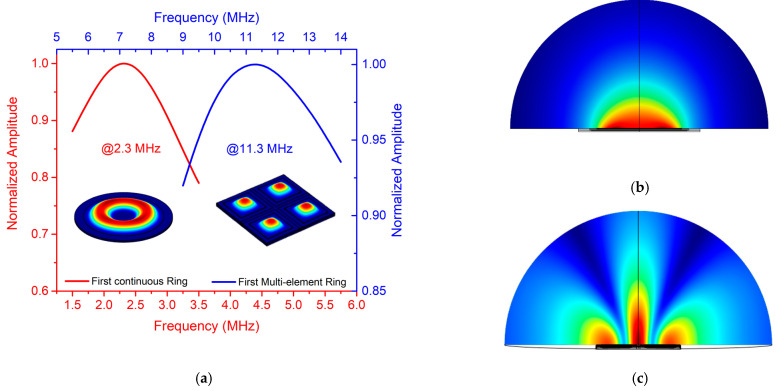
FEM COMSOL dynamic simulation in liquid for the first continuous ring and the first multielement ring: (**a**) frequency response where left-bottom (red curve) corresponds to continuous ring and right-top (blue curve) corresponds to multielement ring; (**b**) continuous ring pressure map at 2.3 MHz; (**c**) multielement ring pressure map at 11.3 MHz.

As the parameter *λij* in Equation (2) and the added virtual mass are only known for defined *d/D* ratios [19,22], we performed some FEM simulations with COMSOL to find the resonance frequencies for the first continuous ring in air and liquid, obtaining 8.9 and 2.3 MHz respectively. The resonance frequency for the continuous ring is 3 times lower than that of the multielement ring (27.8/8.9), which is close to the *f_squared_/f_annular_* ratio when the annular width corresponds to the square PMUT diagonal (see Figure 1 when *l* = *w*). Figure 3 shows the frequency response in liquid for the first ring corresponding to the continuous ring and the multielement ring array. The normalized pressure maps for both rings, considering a propagation medium of 200 μm radius, are shown in Figure 3b,c. The multielement array achieves a higher focus depth in comparison to a continuous ring (considering the same actuated rings) due to its higher operation frequency (see *F_d_* in Table 2), concentrating the acoustic pressure in a narrower beam, but it is affected by side lobes. The focus depth (*F_d_*) is presented in Table 2 for both the multielement ring array device and the continuous concentric ring device considering the same area in both cases. The advantage of focus depth control for the multielement ring array from 67 µm to 1.44 mm is clearly demonstrated.

### 2.2. Acoustic Performance Simulation for the PMUT Array

Acoustic simulations using Field II [23,24] were performed to predict the acoustic performance of the multielement ring array working in liquid (FC-70, c = 685 m/s) at a center frequency of 11.3 MHz (according to the FEM COMSOL simulations shown in Figure 3).

**Figure 4:**
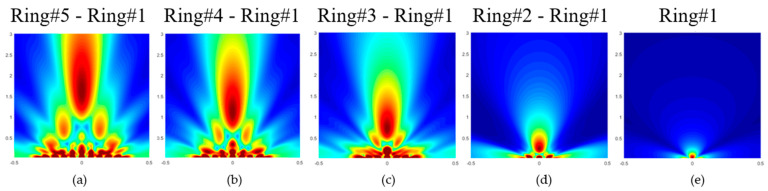
Field II simulated pressure map for the multielement array when transmitting with (**a**) all rings, (**b**) four rings (ring #1 to ring #4), (**c**) three rings (ring #1 to ring #3), (**d**) two rings (ring #1 to ring #2), and (**e**) one ring (ring #1). Axis: Scan in x-direction from −0.5 to 0.5 mm, z-direction from 0.02 to 3 mm.

Figure 4 shows the 2D normalized pressure map from 20 μm to 3 mm along the axial direction and from −500 μm to 500 μm laterally, keeping the array center at (0, 0) coordinates. Note that, as Table 2 shows, by playing with the number of active rings, the focal point can be changed without any extra delay. The beam focusing range from these Field II simulations is a bit higher, reaching 1.6 mm, due to the simulation considering the real element distribution inside the multielement ring array (gap spaces between elements), which is small and is not considered in Equation (1).

**Figure 5:**
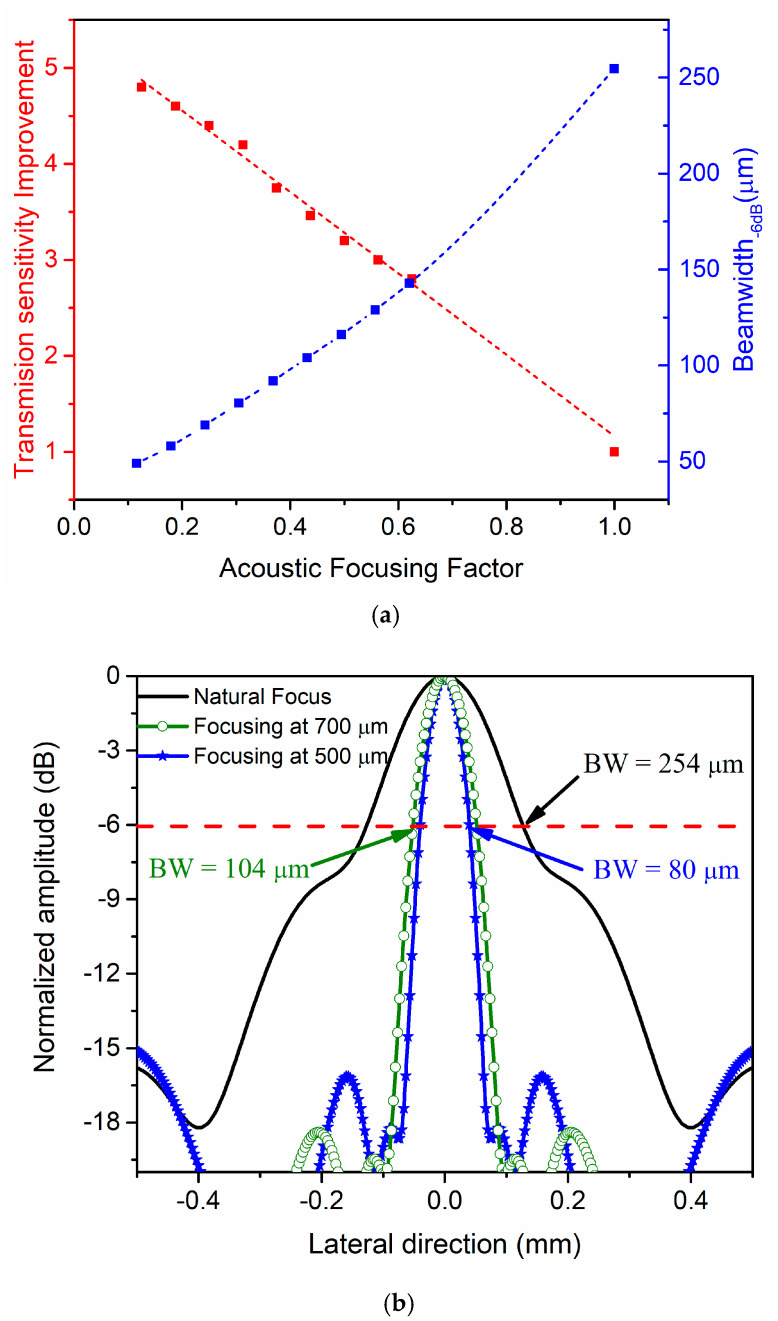
Field II simulations: (**a**) dependence of the transmission sensitivity improving factor (red curve) and the beamwidth (blue curve) on the acoustic focusing factor; (**b**) cross-section in lateral direction at two different focal points (500 and 700 µm, corresponding to acoustic focusing factors of 0.3 and 0.44, respectively).

On the other hand, controlling the applied signal phase, the focal point can also be modified (electronic focusing), achieving higher pressure levels than at the natural focus. In Figure 5a, the red axis shows the dependence of the transmission improvement on the acoustic focusing factor (*Sac*), defined as *Sac* = *Fac*/*N*_0_ where *Fac* is the actual focus and *N*_0_ is the natural focus (1.6 mm). In the simulations, the delays were applied to all the elements that make up each ring. As expected, when the focus point is close to natural focus, there is no transmission sensitivity enhancement (value close to 1), so to ensure at least twice as much the transmission pressure, the acoustic focusing factor should be 0.8 (and consequently the focal point will be 1.3 mm). Furthermore, when it is focused, the acoustic energy is concentrated in narrow beams, decreasing the focus width, and consequently improving the capability to detect small targets. On the other hand, in Figure 5a, the blue axis shows the dependence of the beamwidth at −6 dB on the acoustic focusing factor. In this case, the beamwidth is wider when the acoustic focusing factor is close to 1, as expected.

Figure 5b shows the acoustic pressure field profile along the lateral direction at different focus points, including the natural focus (*N*_0_). As can be seen, the multielement ring array is affected by the generation of unwanted lobes (side lobes). For focus depths greater than 500 µm, these side lobes are below −15 dB, which is proven to be the required dynamic range for imaging [25].

## 3. Experimental Results and Discussion

### 3.1. Electrical Characterization

**Figure 6:**
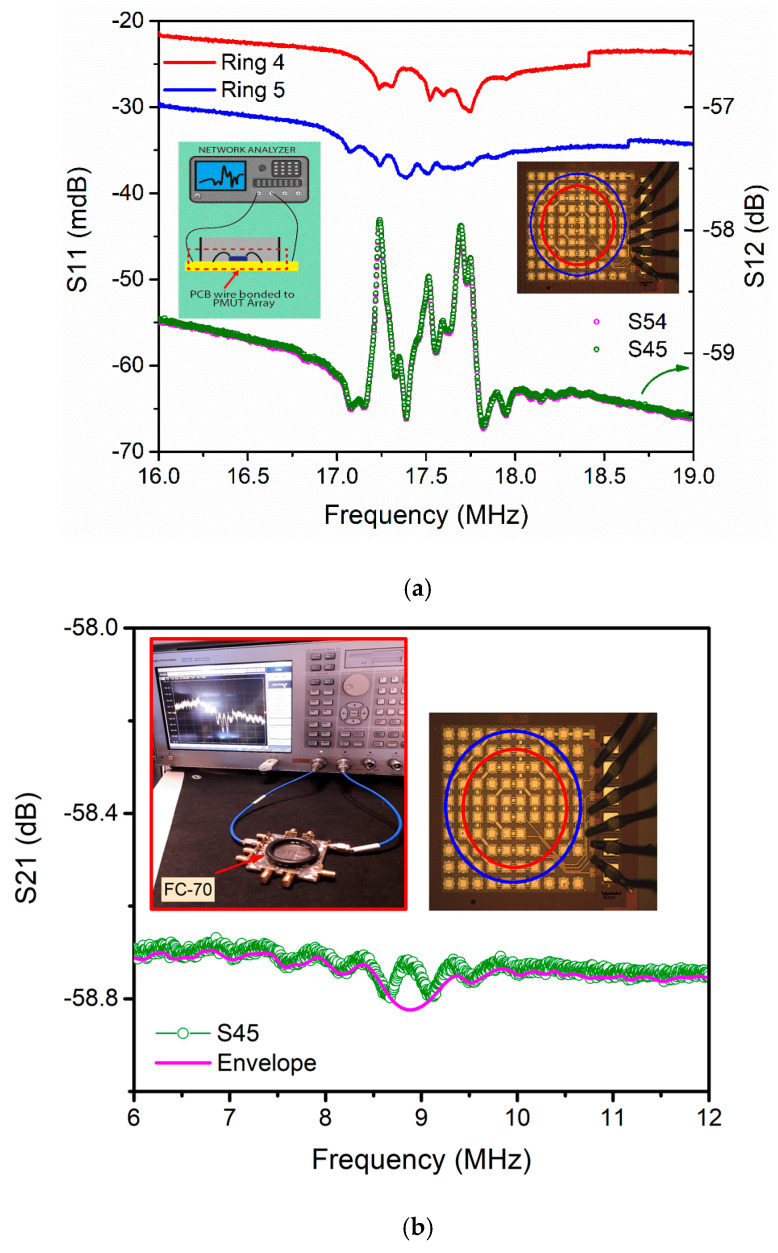
Scattering parameter measurements (**a**) in air (S_11_, red and blue curves with left axis; S_12_, green and rose curves with right axis) using rings #4 and #5 and (**b**) in Fluorinert, S_12_ using rings #4 and #5.

The electrical characterization in the air was done using the multiring PMUTs bonded to a PCB and using a network analyzer (Agilent Technologies, Santa Clara, CA, USA). Figure 6a left inset shows a schematic of the experimental set-up. Each ring was powered with 10 dBm continuous wave to obtain the S-parameters. The frequency response, S_11_, for each ring, gives a center value of 17.5 MHz, which corresponds to the resonance frequency of an individual PMUT (single clamped square PMUT, as reported in [26]). 

In Figure 6a, the red curve shows the frequency response corresponding to ring #4, and the blue curve shows the ring #5 response. The multiple peaks are a consequence of the multiple individual PMUTs forming the ring.

In order to analyze the electrical crosstalk effect in the proposed device, the S_21_ magnitude between pairs of rings was obtained [27]. In Figure 6a, the dotted graph (green curve) corresponds to the measurements between ring #5 and ring #4. The crosstalk level was obtained considering the S_21_ at 19 MHz (out of the resonance peak), giving for this case −59.4 dB which represents 2.1 mVpp (for a 10 dBm input signal). Even if the crosstalk level during the resonance is considered, there would be no significant actuation voltage in the non-actuated rings, decreasing the risk of acoustic interferences. Table 3 summarizes the obtained crosstalk between rings. Note that the lowest level, −77 dB, is between ring #1 and ring #4 and not between the most widely spaced rings (ring #1 and ring #5). This can be attributed to the specific layout of the electrical pads (consecutive pads for rings #1 and #5). The low crosstalk levels (below −56.8 dB) between multielement rings will allow driving each ring independently, ensuring a well-controlled and efficient axial beam-focusing.

The variation of resonance frequencies for each of the individual resonators due to mismatching during the fabrication can be a drawback. This problem can be alleviated when the system is under liquid operation due to the acoustic radiation mass loading effect from the liquid which widens the resonance frequency curve. For a square PMUT transducer under liquid operation in one side, this mass load damping can be quantified according to Equation (5) (where *ρ_liquid_* is the liquid density, *a* is the transducer side, and *µ* is the mass per unit area) [28], which gives *β* = 2.46.

(5) $$\beta =0.342\frac{\rho_{liquid}\times a}{\mu }$$

Then, the expected resonance frequency in liquid will be approximately half the air resonance frequency, i.e., 9.4 MHz (fair/√(1 + β)). Figure 6b shows the electrical frequency response of the crosstalk between ring #5 and ring #4 in FC-70. The resonance peaks appear between 8 and 10 MHz, being in correspondence with the expected value (9.4 MHz). On the other hand, in liquid, the high acoustic radiation damping is translated into a low quality factor and consequently to higher fractional bandwidth, hence lowering the S_21_ magnitude at resonance and smoothing the single-element frequency peaks of the same ring.

### 3.2. Output Pressure Measurements

**Figure 7:**
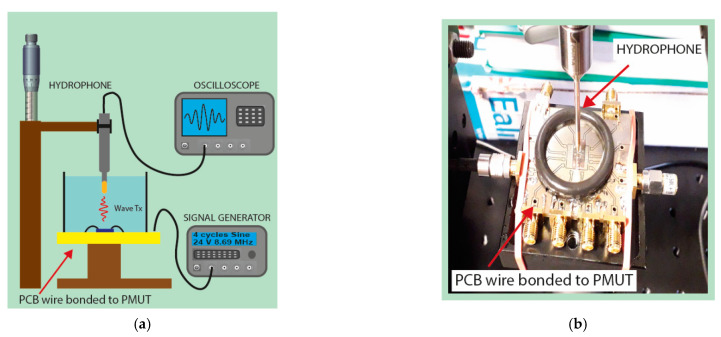
Set-up for the acoustic characterization as actuator in liquid environment (**a**) schematic set-up and (**b**) photo of the experimental set-up.

The multielement ring array was immersed in Fluorinert (c = 685 m/s, *ρ* = 1940 kg/m^3^) and each ring was driven by a signal generator (Keysight, Santa Rosa, CA, USA) with four sine cycles with 24 Vpp. The acoustic pressure was measured with a commercial hydrophone from ONDA (Santa Clara, CA, USA) and displayed in an oscilloscope (Santa Rosa, CA, USA); Figure 7 shows the set-up. The experimental resonance frequency (tuned to maximize hydrophone signal) was 8.7 MHz in accordance with the electrical measurements.

In order to avoid artifacts on the pressure field characterization due to spatial averaging effects (consequence of the influence of the hydrophone’s diameter), there is a limitation on the minimum axial distance between the hydrophone and the ultrasound transducer. Equation (6) should be used [29] to estimate the maximum effective hydrophone radius (*a_h_*), taking into account the transducer radius (*a*_1_), the wavelength in the acoustic media (*λ = c/f*), and the distance between the hydrophone and the transducer (*l*). In our experimental set-up, *a_h_* =100 µm (HNC-0200 radius), *c* = 685 m/s, and *f* = 8.69 MHz, giving the ratio *l/2a*_1_ = 5.06, which means different minimum distances depending on the ring size; Table 4 summarizes these values for all cases (*Lmin* represents *l* in Equation (6)).

(6) $$a_{h}=\frac{\lambda }{8a_{1}}\left(\sqrt{l^{2}+a_{1}^{2}}\right)$$

Therefore, in the experiments, the hydrophone was placed at the distances detailed in Table 4 and raised every 50 µm to obtain the axial pressure at different heights without distortion. These distances are in the far-field, which allows defining the acoustic pressure by Equation (7) [18] (where *R*_0_ is the Rayleigh distance, *P*_0_ is the surface pressure, and *z* is the axial distance).

(7) $$P(z)=\frac{P_{0}R_{0}}{z}$$

The measured points were fitted according to Equation (7), obtaining from the slope the normalized pressure with the distance $NP=P_{0}R_{0}$ (Pa×mm) for each ring (see the results in Table 4). From these measurements, the maximum attainable pressure at the natural focus (~1.2 mm according to Field II simulations at 8.69 MHz) can be computed, evaluating the NP for each ring at 1.2 mm and adding them because, at this point, all acoustic waves are in phase, allowing constructive interference. Finally, the computed total pressure is 50.71 kPa_pp_. This output pressure could be increased until 4.8 times, for example, focusing at 200 µm (see the improvement factor in Figure 5a).

The normalized acoustic pressure (ST) at 1.5 mm from a 1 mm^2^ PMUT array area when it is driven with 1 V was used to compare the multielement ring array with other ultrasound transducers. Taking the previously computed pressure when all rings are excited, 50.71 kPa_pp_, and normalizing with the distance (1.2 mm) and the applied voltage (24 Vpp), we obtain a surface pressure of 2.54 kPa_pp_×mm×V^−1^. According to this and considering the entire area of the multielement ring array (π × (D_5_^2^ − d_1_^2^) = 0.35 mm^2^), the normalized pressure at 1.5 mm is 4.84 kPa/V/mm^2^. Table 5 compares the multielement ring array performance as an actuator with the state-of-the-art approaches, demonstrating a promising performance with a minute area. The normalized output pressure is 55% higher than that reported for arrays of annular AlN flexural rings (2.2 kPa/V/mm^2^ using five-channel ring [6]) and even 92% higher than that for a system for intravascular imaging [2].

In order to complete a deeper characterization of the performance of the system as an actuator and compare the presented multielement array system with a continuous AlN flexural PMUT array [6], the pressure normalized with the area and energy density, (*p*), defined in Equation (8) was computed (where *V* is the applied voltage (24 V), *A_ap_* is the area (0.35 mm^2^), *e*_31,*f*_ is the piezoelectric coefficient (*e*_31,*f*_ = *e*_31_ − υ × *e*_33_ = −1.065 C/m^2^), *λ* is the wavelength (*λ* = *c*/*f* = 79.86 µm), and *p* is the total pressure (50.71 kPa at 1.2 mm)).

(8) $$\bar{p}=p\times \frac{\lambda }{V\times A_{ap}\times e_{31,f}}$$

Applying this expression at 1.2 mm (the natural focus) when the multielement five-ring array is used, a normalized pressure per area per energy density of 453 kPa×mm^−2^/J×cm^−3^ is obtained, which is 2.5× better than that of the five-channel continuous ring array without delays presented in [6] (184 kPa×mm^−2^/J×cm^−3^). Compared with the result when the same system is focused at 1.9 mm (588 kPa×mm^−2^/J×cm^−3^, [6]), our multielement ring array still exhibits a competitive value and will allow higher pressure output at shorter focus depths if some phase-beam focusing is used.

### 3.3. Pulse-Echo Measurements

**Figure 8:**
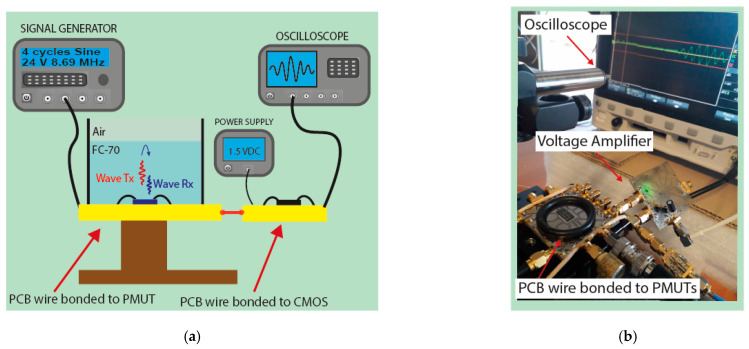
(**a**) Schematic set-up for pulse-echo measurements; (**b**) photo of the experimental set-up.

Thanks to its reduced area, the multielement ring array can be a candidate for catheter-based ultrasound imaging; consequently, it was also characterized as a pulse-echo acoustic system. In this case, the central ring was used as a receiver, and the rings #5, #4, and #3 were used as transmitters. The same signal generator was used to drive the transmission with four sine cycles at 8.69 MHz with 24 Vpp. The receiving ring was externally connected to an integrated CMOS voltage amplifier with a gain of 25 dB [31]. The liquid thickness over the PMUT chip was tuned to obtain different times of flight (ToFs) and consequently different acoustic paths (*AP = ToF × c*) giving a round trip from 1 to 5 mm; Figure 8 shows the set-up.

**Figure 9:**
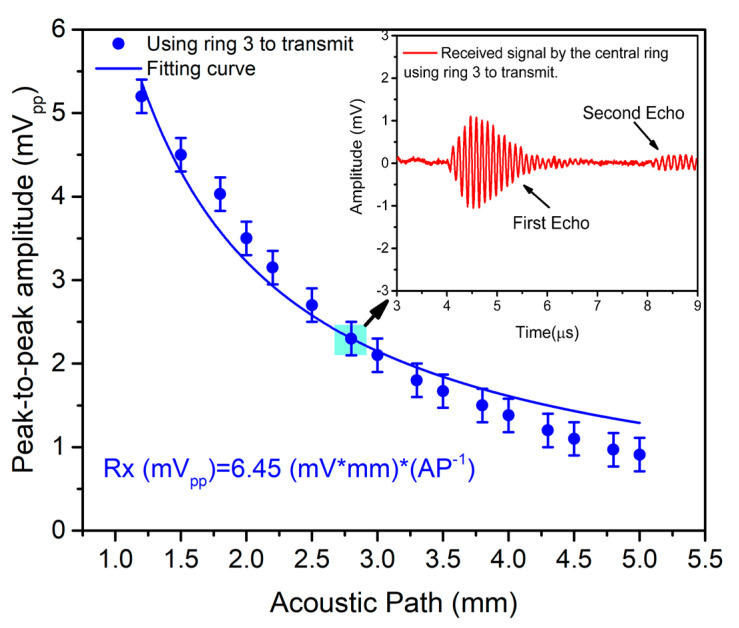
Pulse-echo experiment with ring #3 as transmitter and ring #1 as receiver. The received signal is plotted as a function of the acoustic path, *AP*, using FC-70-air interface as reflecting surface. Error bar = ±200 µV. Inset: Time-domain signal at 2.8 mm round trip.

Figure 9 shows the measured echo voltage and its dependence on the acoustic path using ring #3 (blue points) (same measurements were also acquired for ring #4 and ring #5). Taking the fitting results, the receiving sensitivity (SR) can be computed as *Rx/Pt* (where *Pt* is defined by the normalized pressure, NP/z, in Table 4), giving an average value of 441.6 nV/Pa ((Rx_central_/Pt_3_ + Rx_central_/Pt_4_ + Rx_central_/Pt_5_)/3). This receiving sensitivity is affected by the parasitic capacitances between the PMUT and voltage amplifier (PCB, connectors, cables, etc.) according to Equation (9) [32]:

(9) $$SR=SR_{EOC}\times G\times \frac{C_{centralRing}}{C_{centralRing}+C_{inLNA}+C_{parasitic}}$$

where *SR_EOC_* is the “end-of-cable open-circuit sensitivity”; *G* is the amplifier gain (25 dB); *C_centralRing_* is the receiving element capacitance (254.9 fF extracted from COMSOL); *C_inLNA_* is the voltage amplifier equivalent capacitance (609 fF) [16]; and *C_parasitic_* is the parasitic capacitance associated with PCBs, connectors, cables, etc. (at 6.5 pF). Hence, the intrinsic sensitivity (*SR_EOC_*) for the smaller inner ring is 717.8 nV/Pa. This value is very competitive in comparison with some commercial hydrophones with sizes comparable to our ring #1 (diameter of ~149 μm) but with smaller nominal *SR_EOC_* pressure sensitivities: HNC-0200 from ONDA (200 μm diameter, SR_EOC_ = 28 nV/Pa [32]) or NH0200 from Precision Acoustics (200 μm, 55 nV/Pa with amplifier [33]).

**Figure 10:**
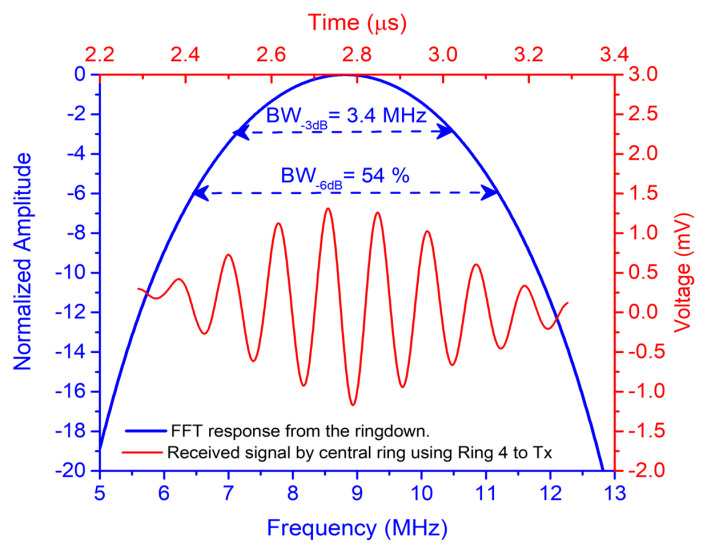
Acoustic pulse-echo measurement using ring #4 to transmit and central ring to receive (FC-70 thickness at 800 μm). Red curve (top-right red axis): time-domain response. Blue curve (left-bottom blue axis): FFT from the ringdown.

The time-domain response shown as the red curve in Figure 10 corresponds to pulse-echo measurement when ring #4 is used to transmit and the central ring is used to receive. The time-of-flight of the received echo is 2.34 μs, which gives an FC-70 thickness of 800 µm (FC70_thickness_ = ToF × c/2). Taking the ringdown, the fast Fourier transform was computed (see Figure 10, blue curve), giving a resonance frequency (f_0_) of 8.8 MHz with fractional bandwidth at −6 dB close to 54%.

**Figure 11:**
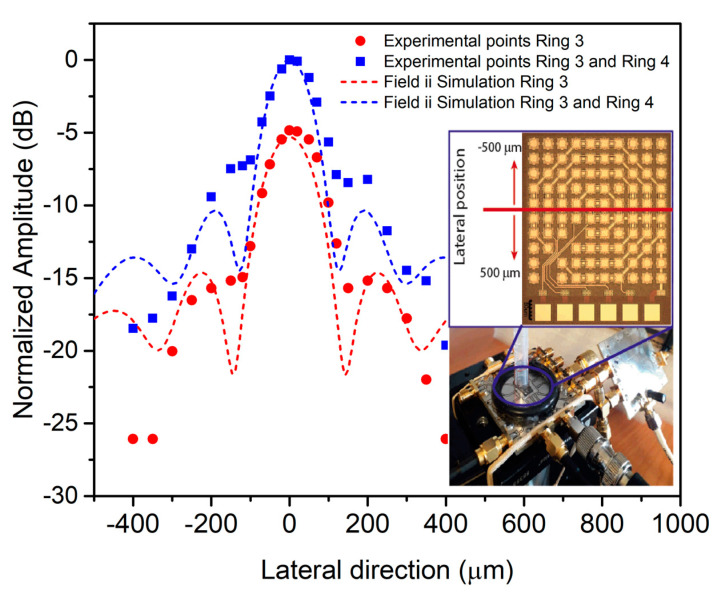
Simulated and experimental behavior along the lateral direction at 790 µm axial distance. Red curves correspond to ring #3 as transmitter and blue curves correspond to ring #3 + ring #4 (dots: experimental points; lines: Field II simulations). Inset: Experimental set-up.

The acoustic beamwidth was determined through a pulse-echo experiment where ring #3 and ring #4 were used as transmitters and the central ring was used as the receiver. A 150 µm diameter conductive wire was used as a reflecting surface and was placed at 790 µm over the array’s surface. The wire covered all PMUTs in one lateral direction, and in the other direction, it was mechanically displaced 500 µm to each side from the center of the array (see Figure 11 inset). Figure 11 provides experimental and simulation results (using Field II) for when only ring #3 or ring #3 + ring #4 were excited. According to the simulations, the beamwidth at −6 dB is around 160 µm when ring #3 + ring #4 are used (blue lines in Figure 11) and is 180 µm when only ring #3 is used (red lines in Figure 11). Moreover, the maximum amplitude increases by about 6 dB when both rings are used. The experimental points demonstrate a very good agreement with the simulated ones.

### 3.4. Focusing Capabilities

The focusing capabilities were demonstrated through a pulse-echo experiment using ring #3 and ring #5 as transmitters and the central ring as a receiver (see set-up in Figure 8). The acoustic path was tuned in the same way, changing the FC-70 thickness and measuring the time-of-flight corresponding to ring #3 (nearest to receiver element).

**Figure 12:**
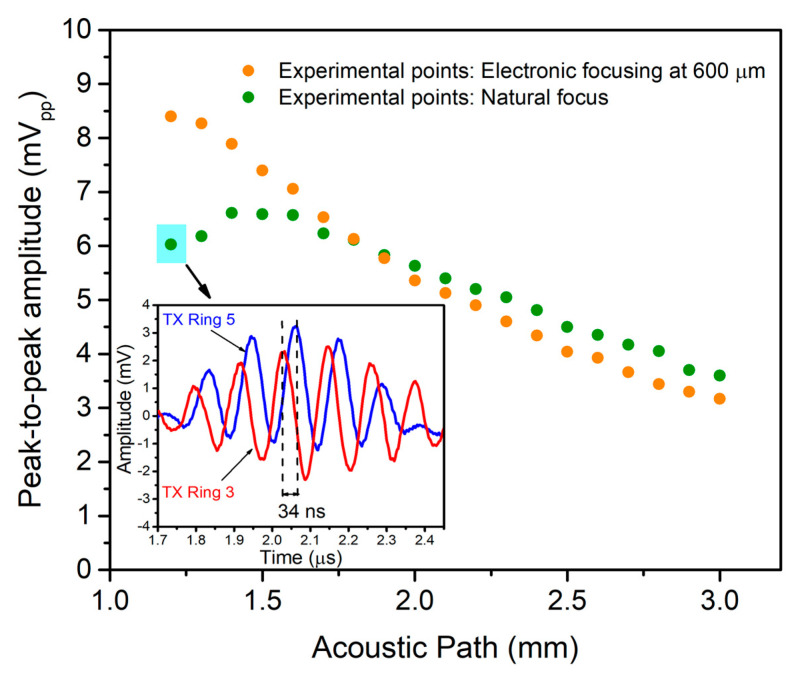
Voltage measured by the central ring when ring #3 and ring #5 are driven at the same time (green points) or electronically focused (orange points). Inset: Time-domain response of the echo signal from ring #3 and ring #5 to determine the needed time delay (34 ns).

Figure 12 (inset) shows the signal received by the central ring when ring #3 and ring #5 are used separately. As can be seen, the received acoustic waves from ring #3 and ring #5 are not in phase; in consequence, when both rings are driven together, the total acoustic pressure is less than the sum of both echoes. The behavior without any delays is shown in Figure 12 (green points), giving a maximum level of 6.59 mVpp close to 1.5 mm round trip (natural focus). Considering this value and the computed receiving sensitivity (441.6 nV/Pa), the pressure on the array’s surface is 14.9 kPa_pp_.

For electronic focusing or phased-array rings, 34 ns was applied to ring #3 during transmission to allow both acoustic waves to arrive in phase, achieving a maximum acoustic pressure at 0.6 mm (see Figure 12, orange points). Computing the focusing improvement factor as the ratio between maximum received echoes (8.4 mVpp/6.59 mVpp), a 1.3× improvement factor is obtained, which is translated into an acoustic pressure of 19.4 kPa_pp_ (1.3 × 14.9 kPa_pp_).

### 3.5. 1D Line-Scan Imaging

**Figure 13:**
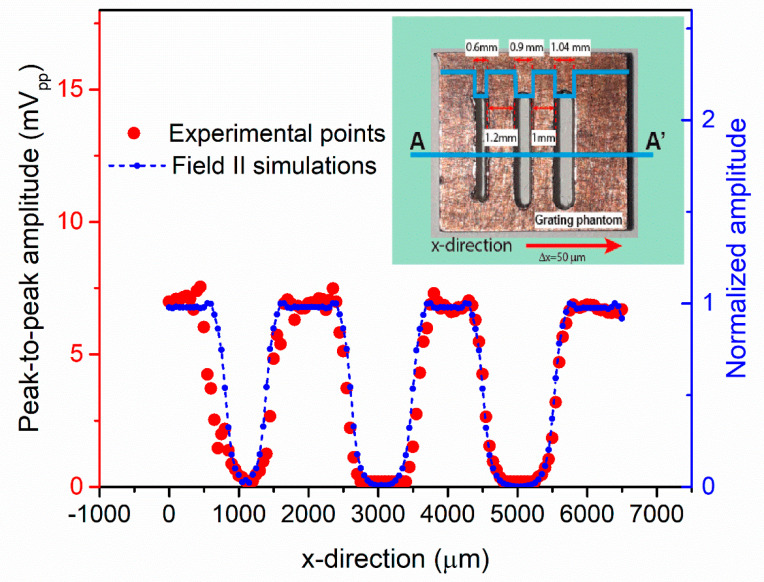
Pulse-echo ultrasonic 1D image of a grating phantom. Red curve: experimental points; blue curve: Field II simulations. Inset: Grating phantom with A-A’ scanned profile.

The imaging capability was tested using a Cu grating phantom with three holes with different widths (600, 900, and 1040 µm) and gaps (1.2 and 1 mm) between them (see Figure 13 inset). The sample was immersed in FC-70 and placed at 790 μm on top of the multielement ring array. A micrometric system was used to displace it along the x-direction (perpendicular to the holes) with steps of 50 μm, while the y-direction was fixed at the sample’s center. Two rings (#3 and #4) were driven with four cycles at 8.69 MHz with 24 Vpp, and the central ring (connected to the CMOS voltage amplifier as before) was used to detect the reflected echoes (see the set-up in Figure 8).

Figure 13 (red points) show the experimental peak-to-peak amplitudes received by the central ring, giving a maximum value of around 7.5 mV_pp_ and clearly reproducing the AA’ profile with the three holes.

On the other hand, a scatter phantom close to the grating was modeled in Field II with the purpose of obtaining the scanning pattern under the same assumptions. The normalized signal received by the central ring when rings #3 and #4 are excited is shown in Figure 13 (blue points), demonstrating a good agreement with the experimental one and validating the capability to perform acoustic imaging with the multielement ring PMUT presented.

## 4. Conclusions

This paper presents a multielement ring ultrasound transducer array based on AlN PMUTs fabricated with a MEMS-on-CMOS process. The presented multielement ring array eliminates the dependence of acoustic wave frequency on the diameters of the annular array and achieves accurate control of the focus depth (from 67 µm to 1.4 mm), which is 4.9 times greater than that of the equivalent continuous ring array. The low crosstalk between different rings (levels under −56.8 dB) allows it to be used in modern ultrasound applications where the maximum of the ultrasound beam must be controlled efficiently in the axial direction. The PMUT-based ring array, with a very reduced area, generates high pressure levels (4.84 kPa/V/mm^2^ at 1.5 mm) at 8.7 MHz in a liquid environment, being very competitive with other annular arrays using bulk piezoelectric, CMUT, or PMUT fabrication approaches. The pulse-echo experiments with a voltage amplifier (gain of 25 dB) externally connected to the central ring gave a receiving sensitivity of 441.6 nV/Pa, which could be increased around 700 nV/Pa when the PMUT is monolithically integrated on the CMOS circuitry. The 1D imaging test through mechanical scanning demonstrates the possibility to obtain high-performance ultrasound imaging systems. With this performance and considering its small size (below 1 mm^2^), the presented multielement ring array fabricated with a PMUT-on-CMOS technology becomes an interesting ultrasound transducer for applications in which size, cost, reliability, and performance are a must, such as wearables and catheter-based systems. Greater focal depth and output pressure can be achieved at the same frequency by increasing the number of rings within the same technology.

## Figures and Tables

**Table 1:** Material properties and thickness used in FEM simulations.

PMUT Layer	Properties			Geometric	
	Mat.	Young’s Modulus (GPa)	Density (kg/m^3^)	Side (µm)	Thick. (µm)
Substrate	SiO_2_	70	2200	60	2
Bottom Elec.	Al	70	2700	46	0.4
Piezoelectric	AlN ^1^	279	3230	60	1.3
Top Elec.	Al	70	2700	28.3	0.35
Passive	Si_3_N_4_	250	3100	60	1.5

^1^ The piezoelectric coefficients e_33_ and e_31_ used in COMSOL are 1.55 C/m^2^ and −0.6 C/m^2^ respectively.

**Table 2:** Focus depth comparing multielement ring array with an equivalent continuous ring array using Equation (1) and the resonance frequencies obtained from the COMSOL simulations. The *F_d_* is calculated with all actuated smaller rings. (c = 685 m/s).

Number of Rings	Dimensions		Focal Point → Fd (μm)	
	D (μm)	d (μm)	Continuous Ring; f = 2.3 MHz	Multielement Ring; f = 11.3 MHz
1	74.28	17.68	13.7	67.4
2	139.28	82.68	50.3	247.3
3	204.28	147.68	109.2	536.6
4	269.28	212.68	190.4	935.4
5	334.28	277.68	293.8	1444

**Table 3:** Crosstalk between rings, according to S21 experimental magnitude at 19 MHz in air.

dB	1	2	3	4	5
1	×	−56.8	−76	−77	−70
2		×	−69.5	−74	−72.5
3			×	−62	−69.5
4				×	−59.4
5					×

**Table 4:** Individual ring characterization as actuator in FC-70 using HNC-0200 from ONDA (at 8.69 MHz). *Lmin* is computed from Equation (6); *NP* is experimentally measured.

Ring	*Lmin* (mm)	*NP* (kPa×mm)	Pressure (kPa) at 1.2 mm
1	0.531	6.33	5.28
2	1.19	10.04	8.37
3	1.84	14.92	12.43
4	2.2	15.41	12.84
5	3.16	14.2	11.79

**Table 5:** Comparison of ring arrays with different technological approaches.

Parameters	[30]	[2]	[6]	This Work
	2011	2018	2019	
Transducer technology	CMUT	PZT matrix	AlN PMUT	AlN PMUT
Configuration	Multielement ring	Multielement ring	Continuous ring	Multielement ring
Medium	Vegetable oil	Water	Mineral oil	FC-70
Frequency (MHz)	1.2	14	6	8.69
Area (mm^2^)	12.76 ^1^	6.28 ^1^	7.07 ^1^	0.35
Pressure (kPa/V@mm)	13.2	0.4	2.8	2.11
	@1.5 ^2^	@6	@5.4	@1.2
NP (kPa×mm/V)	19.8	2.4	15.2	2.54
ST (kPa/V/mm^2^)	1.11 ^3^	0.38 ^3^	2.18 ^3^	4.84

^1^ Computed considering the transducers’ dimensions. ^2^ Taking the peak-to-peak pressure (2 × 66 kPa) and the applied voltage (10 V). ^3^ Computing as the ratio between NP evaluated at 1.5 mm and the area.

## Data Availability

Not applicable.
